# Selection of a quality of life instrument for polio survivors in Northwest Nigeria

**DOI:** 10.1186/s12955-020-01552-4

**Published:** 2020-09-21

**Authors:** Surajo Kamilu Sulaiman, Heather Michelle Aldersey, Vincent G. DePaul, Bashir Kaka

**Affiliations:** 1 School of Rehabilitation Therapy, Queen’s University, Louise D. Acton Building, 31 George Street, Kingston, Ontario K7L 3N6 Canada; 2 Department of Physiotherapy, Faculty of Allied Health Sciences, College of Health Sciences, Bayero University, Kano, PMB 3011 Nigeria

**Keywords:** Quality of life, Outcome measure, Questionnaire, Psychometric properties, Cultural relevance, Translation, Adaptation

## Abstract

**Background:**

To generate high-quality evidence, contextually relevant outcome measurement instruments are required. Quality of life evaluation among polio survivors typically involves the use of generic instruments, which are developed and validated among a different groups of people. There is no clear evidence whether these instruments are appropriate for the measurement of quality of life among polio survivors in northwest Nigeria. The purpose of this review is to identify and select a pre-existing instrument that is best suited for the measurement of quality of life among polio survivors in northwest Nigeria.

**Methods:**

Using the findings of a previous scoping review of the literature and qualitative descriptive study, we screened 11 quality of life instruments that are used in polio literature. We identified and selected the most appropriate instrument, which reflected the perspectives of polio survivors in northwest Nigeria and at the same time exhibited good measurement properties.

**Results:**

The Quality of Life Index, World Health Organization Quality of Life Brief, and Comprehensive Quality of Life Scale are consistent with the perspectives of polio survivors in northwest Nigeria and have satisfactory measurement properties. Among these instruments, the Quality of Life Index satisfied most of the screening criteria we employed and is suitable for cross-cultural adaptation in northwest Nigeria.

**Conclusion:**

Most instruments that are employed to evaluate the quality of life of polio survivors were not primarily designed as a measure of quality of life. To select the appropriate instrument, there is a need to consider and reflect the perspectives of the individuals, to improve the validity of the measurement.

## Introduction

Paralytic polio is a neuromuscular disorder, which is characterized by acute flaccid paralysis, especially in the upper and lower limb muscles, resulting from the destruction of motor neurons in the brainstem and spinal cord by poliovirus [2, 10]. Evidence has shown that secondary complications in the form of post-polio syndrome and other related neuromuscular impairments are common among polio survivors, which could lead to deterioration in health and functional status of the individual [39, 40, 44, 57]. Globally, about 20 million people are living with varying degrees of polio-related disability [25]. Despite the dearth of accurate statistical information, extant literature suggests that there are approximately 15 polio survivors per 100,000 people in Nigeria [34]. Although Nigeria has crossed 3 years without a new case of a wild poliovirus, it is yet to be officially declared polio-free by the World Health Organization [63]. Thus, Nigeria is likely to have a significant proportion of the over 1 million polio survivors in the African continent [17, 25].

Studies have shown that in comparison with the general population, polio survivors frequently report poor health and quality of life (QoL) [1, 21, 27, 51, 64]. This finding is especially common for individuals who are living in countries with limited social and infrastructural resources such as Nigeria [25]. Polio survivors are unique in their life experiences as they grow older. They are likely to experience further disability as a result of reemerging impairments such as post-polio syndrome and other socio-environmental barriers [25, 46]. Therefore, the measurement of QoL among polio survivors needs to take these experiences into cognizance. Moreover, QoL evaluation in individuals with a chronic condition like polio survivors typically presents with some challenging issues as a result of a phenomenon called response shift [20, 22]. The response shift phenomenon refers to a change in the self-evaluation of QoL as a result of change in the respondent conceptualization of QoL, values, or internal standards of measurement [49]. Thus, the response shift could significantly alter the validity and reliability of QoL measurement overtime. Hence, it is, therefore necessary to integrate response shift when evaluating the QoL of polio survivors, in order to account for a true change in their QoL.

QoL is an important outcome that can be employed to evaluate the unmet social and healthcare needs of individuals and also determine the success of various interventions [28, 37, 43, 48, 52]. Measurement of QoL among polio survivors can also provide a person-centered approach to evaluate the effects of paralytic polio on the physical, social, and psychological wellbeing of the individual [1, 5, 21, 51, 64]. Within polio literature, QoL studies are typically reported from high-income countries that eradicated polio decades ago. Studies from low-income countries, especially where polio cases are common, such as Afghanistan, Nigeria, and Pakistan are scantily reported [56]. Hence, there is a need for more empirical evidence from low-income countries like Nigeria, to provide a holistic understanding of the wellbeing of polio survivors. To assess QoL using reliable and meaningful scientific investigation, contextually relevant and validated measurement instruments are necessary [41, 58].

Development and validation of a new QoL instrument is exceptionally laborious. Hence, researchers suggest cross-cultural adaptation and validation of pre-existing instruments [18]. One of the major challenges of this process is how to select the most appropriate instrument. There is a lack of robust guidelines for the selection of QoL instruments for cross-cultural adaptation [8, 32]. However, when selecting the outcome instruments, users tend to evaluate the conceptual and measurement properties of the scales, to ascertain their relevance and validity in the intended population [8, 13, 32, 47]. We could not identify any QoL instrument that was developed specifically for polio survivors. Evidence from the literature shows that generic QoL scales are typically employed by users to examine the QoL of polio survivors in Nigeria, based on the feasibility of the instruments, specifically the client’s comprehensibility and mode of administration [1, 36].

**Fig. 1:**
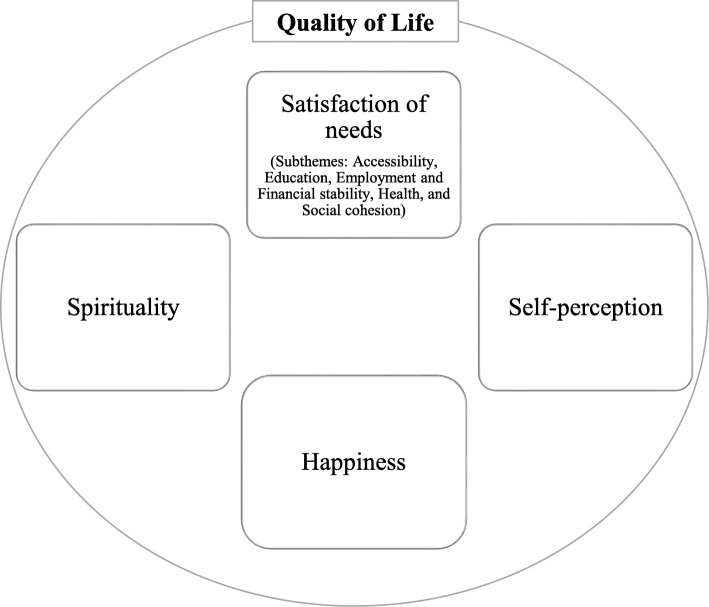
Perception of quality of life of polio survivors in northwest Nigeria

In our previous review, we identified 11 instruments that have been employed to measure the QoL of polio survivors globally [56]. Table 1 provides information about these instruments. Further, polio survivors in northwest Nigeria characterized QoL as a reflection of four major themes: satisfaction of needs, happiness, spirituality, and self-perception. Under the satisfaction of needs, polio survivors described the satisfaction of the following needs: accessibility, education, employment and financial stability, health, and social cohesion. Besides, the polio survivors expressed self-perception as comprising self-value/self-worth, physical/bodily appearance, and feeling independent [55]. Figure 1 provides a pictorial organization of the themes illustrating the perception of the QoL of polio survivors in northwest Nigeria. This review utilizes the extant literature to identify and select a QoL instrument(s) that is amenable for cross-cultural adaptation in the Nigerian context. We aimed to identify and select an instrument that has good psychometric properties and at the same time reflects the perspectives of polio survivors in northwest Nigeria. Our guiding research question was: which pre-existing instrument is best suited for the measurement of QoL among polio survivors in northwest Nigeria?

**Table 1:** Quality of life instruments used in polio literature

Instrument	Number of Items	Domains
Short-form 36 (SF-36)	36	Physical Functioning, Role physical, Bodily pain, General Health, Vitality, Social functioning, Role Emotional, and Mental Health
Nottingham health profile (NHP)	38	Physical mobility, Social isolation, Emotional reactions, Pain, Sleep, and Energy
Quality of life index (QLI)	35	Health and functioning, Social and economic, Psychological and Spiritual, and Family
EuroQol-5D (EQD5)	5	Mobility, Self-care, Usual activity, Pain/Discomfort, and Anxiety/Depression
Kaasa’s questionnaire (KQ)	12	Psychosocial well-being, Medical side-effects, Activities of daily living and Physical performance
Quality of life profile (QP)	44	Life-picture, Life-areas, Problems and Acceptance
World Health Organization quality of life questionnaire- Brief (WHOQOLBREF)	26	Physical health, Psychological health, Social relationships and Environmental
Comprehensive quality of life scale (CQS)	35	Material well-being, Health, Productivity, Intimacy, Safety, Place in community and Emotional well-being
Satisfaction with life scale (SWLS)	5	Not Applicable
Swedish health-related quality of life questionnaire (SWED-QUAL)	61	Physical functioning, Role functioning, Emotional well-being, Pain, Sleep, Family functioning and General health perceptions
Short-form 12 (SF-12)	12	Physical Functioning, Role physical, Bodily pain, General Health, Vitality, Social functioning, Role Emotional, and Mental Health

## Method

**Fig. 2:**
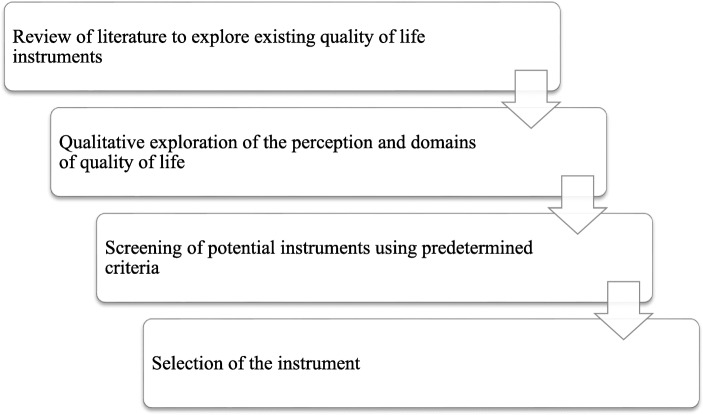
Flow chart illustrating the selection process of the instrument

We conducted this integrative review using the findings of our previous studies: a scoping review of QoL assessment instruments among polio survivors [56] and a qualitative exploration of the perception and domains of QoL for polio survivors in northwest Nigeria [55]. We employed pre-existing recommendations to select the most appropriate QoL instrument(s) that reflects the perspectives of polio survivors in northwest Nigeria and at the same time possesses good psychometric properties. We screened the available QoL instruments by evaluating the following characteristics, which we adapted from the literature: the intent of the instrument, content suitability, measurement properties, feasibility, and considerations in adapting the instrument for cross-cultural use [8, 13, 32, 47]. These characteristics provide a relevant and critical consideration for the selection of appropriate outcome measurement instruments. Our approach to selection of a suitable QoL instrument was inspired by the recommendations of the Consensus-based Standards for the Selection of Health Measurement Instruments (COSMIN) practical guidelines for the selection of an outcome measurement instrument [47]. Figure 2 illustrates the process we followed in the selection of the instrument(s).

### Screening of potential instruments by evaluating their characteristic features

The screening of the instruments was done by two authors (SS and BK) to minimize bias and improve validity. We evaluated the characteristics of the instruments using the guidelines proposed by Bentzen and colleagues [8], and where necessary, we modified the taxonomy and definition of the feature based on the COSMIN consensus [41, 47]. To make our final instrument selection decision, we evaluated the intent of the instrument, content suitability, measurement properties, feasibility, and considerations in adapting the instrument for cross-cultural use. These properties are non-hierarchical, equally relevant, and constitute an integral part of the selection process.

### Intent of the instrument

The intent of the instrument refers to the aim of the instrument, which addresses the question: for what purpose was the instrument developed? It reflects the appropriateness of the instrument and the construct that the instrument purports to measure [7, 8, 24, 32]. We identified the intended purpose of each of the QoL instruments from our scoping review, using the developers’ description of the aim of the instrument, which we presented verbatim. We intended to select an instrument(s) that was solely developed for the measurement of QoL.

### Content suitability

Content suitability denotes the extent to which the instrument reflects the perspectives of the target individuals. For this review, we looked for reflection of the perspectives of polio survivors. Content suitability mirrors some aspects of validity since it capitalizes on the representation of the construct based on the individuals’ perceptions [7, 24]. We evaluated the content suitability of each of the instruments by linking the instrument to our qualitative findings. We identified the domains and contents of the instruments from our scoping review and subsequently mapped them to the four fundamental perceptions of QoL and their aspects (satisfaction of needs, self-perception, spirituality, and happiness) as identified by the polio survivors in northwest Nigeria who participated in our qualitative study [55]. Specifically, we linked the contents of the instruments, based on their face validity and consistency, to the perception of QoL of the polio survivors. We intended to identify and select an instrument that mirrors all the perspectives of polio survivors in northwest Nigeria. Table 2 provides a checklist of the concepts we employed and their definitions, while Table 3 illustrates how an appraiser applies the checklist to link the contents of the instruments to the perspectives of the polio survivors.

**Table 2:** Check list and definitions of concepts

Checklist/Concept	Definition*
Satisfaction of needs	Refers to the fulfilment of fundamental life needs that are necessary for flourishing, specifically, accessibility, education, employment, financial stability, health, and social cohesion.
• Accessibility	• *Refers to the ease of access to the built environment and transportation system.
• Education	• *Refers to the acquisition of formal education and religious (Islamic) theology.
• Employment	• *Refers to a gainful employment or paid job that can provide the individual with the necessary financial support to live independently.
• Financial stability	• *Refers to a state of financial buoyancy or sufficiency.
• Health	• *Refers to a state of being healthy physically, mentally, and spiritually.
• Social cohesion	• *Refers to social belongingness and community and family relationships.
Self-perception	*Refers to self-value, physical appearance, and feeling independent (i.e. the ability to perform activities of daily living).
Spirituality	*Refers to religious faith and religious practices.
Happiness	*Refers to the state of being joyous, cheerful, calm and contented with life offerings.

All definitions are based on the findings of our previous qualitative study [55]

**Table 3:** Application of the checklist

**How to apply the checklist**	
Step 1: Identify the main meaningful unit within the items of the instrument	
Step 2: Check if the meaningful unit is consistent with any of the available concepts	
Step 3: Link the meaningful unit to the most appropriate concept base on face validity	
Step 4: Compute the total number of items in the scale that are link to each concept	
**Example of how an appraiser applies the checklist (using item 2 from WHOQOLBREF)**	
Item 2: How satisfied are you with your health?	
Step 1: Main meaningful unit = Satisfaction with health	
Step 2 & 3: Satisfaction with health = Consistent with ‘Satisfaction of needs (health)’	
Step 4: Number of item = 1	

The protocol of the application of the checklist is derived from the work of [12]

### Psychometric analysis

Psychometric analysis refers to the evaluation of the measurement properties of the instruments, based on evidence of reliability (internal consistency, reliability, measurement error), validity (content validity, construct validity, criterion validity, cross-cultural validity), and responsiveness [42]. We assessed the reported psychometric properties of the instruments using a modified criteria described by [47, 60]. These criteria were derived from the COSMIN checklist for assessment of psychometric properties of outcome measurement instruments [47]. We rated the reported measurement properties of each of the instruments as adequate (i.e. good/positive) when the reported scores of the property are consistent with our criterion, inadequate (i.e. poor/negative) when the scores of the property are below our criterion, and not available when the property was not reported. We made this judgement based on the criteria for good measurement properties described by the COSMIN practical guideline for the selection of outcome measurement instruments [47]. The details of how we applied the criteria were reported in our previous scoping review [56]. Table 4 illustrates the criteria we followed in evaluating the measurement properties. We intended to identify and select instrument(s) with evidence of good measurement properties.

**Table 4:** Psychometric evaluation

**Internal consistency**	We rated internal consistency as adequate when the Cronbach alpha was ≥0.7, not adequate when the criterion was not met and not applicable (NA) when internal consistency was not reported.
**Reliability**	We considered reliability as adequate when the Intraclass Correlation Coefficient (ICC) or weighted Kappa was ≥0.7, not adequate when the criterion was not met and not applicable (NA) when reliability was not reported.
**Content validity**	We considered content validity as adequate when the target population was involved in the development of the scale, not adequate when the criterion was not met and not applicable (NA) when the property was not reported.
**Construct validity** *Structural (factor analysis)*	We rated construct validity as adequate when the factors explained at least 40% of the variance, not adequate when the criterion was not met and not applicable (NA) when construct validity was not reported.
*Hypothesis testing*	We rated construct validity as adequate when the correlation with an instrument measuring the same construct was ≥0.50 (OR at least 75% of the results were in accordance with the hypotheses) AND correlation with related constructs was higher than with unrelated constructs. Not adequate when the criterion was not met and not applicable (NA) when construct validity was not reported.
**Criterion validity**	We rated criterion validity as adequate when the correlation with a criterion instrument(s) was ≥0.50, not adequate when the criterion was not met and not applicable (NA) when criterion validity was not reported.
**Measurement error**	We rated measurement error as adequate when the Smallest Detectable Change (SDC) or Limit of Agreement (LoA) was less than Minimal Important Change (MIC), not adequate when the criterion was not met, and not applicable (NA) when measurement error was not reported.
**Responsiveness**	We rated responsiveness as adequate when the correlation with the anchor instrument was ≥0.50 (OR at least 75% of the results were in accordance with the hypotheses), not adequate when the criterion was not satisfied and not applicable (NA) when responsiveness was not reported.

### Feasibility

Feasibility refers to the burden, time, and resources required to administer, score, analyze, and interpret the instrument. It includes aspects such as comprehensibility, interpretability, cost of the instrument, completion time, mode of administration, etc. [47]. We assessed user comprehensibility using the number of meaningful units we identified within the instruments. A meaningful unit refers to a meaningful concept, which contains words, phrases, or sentences that are related to each other through their content and context [23]. We considered the instrument user-comprehensive when the number of meaningful unit was equal to or greater than the number of items of the instrument. We assessed the interpretability of the instruments based on how scoring is interpreted. We reported whether the instrument has a meaningful domain score (profile), aggregate scores (indices), or both. For the cost of the instrument, mode of administration, and completion time, we evaluated these features based on the developers’ information about the instruments. We intended to identify and select instrument(s) that was comprehensible, interpretable, inexpensive, self or user-administered, and requires moderate time to complete.

### Considerations in adapting the instrument for cross-cultural use

Considerations in adapting the instrument for cross-cultural use deals with the core aspects of translation and adaptation of the instrument into other cultures [8]. To administer instruments in another culture, there is a need for cross-cultural adaptation and if this is done, the user needs to be confident about the richness and the rigor of the translation and adaptation process [8, 18]. The major requirement in any cross-cultural adaptation is the retention of equivalence, which includes conceptual, item, semantic, operational, and measurement [29, 30]. We could not assess equivalence since the process requires translation of the instruments. However, we evaluated cross-cultural relevance of the contents of the instruments based on their face validity. We assessed whether the contents of instruments could attain item relevance in Hausa culture by exploring the domains and items of the instruments and comparing them with the perception of QoL among polio survivors in northwest Nigeria. We also examined the meaning of the items of the instruments to determine whether they could attain semantic relevance in the Hausa language. Finally, our intention was to identify and select an instrument(s) that demonstrates item and semantic relevance.

## Results

### Screening of instruments

Table 5 provides details of the screening performance of the instruments. The following explains the key outcomes of the screening of the instruments.

**Table 5:** Screening performance of the instruments

Instrument	Intent of the instrument	Content suitability (reflection of polio survivors’ perspectives)	Psychometric properties	Feasibility	Cross-cultural relevance (item & semantic relevance)
Short-form 36 (SF**-**36)	Health status measure	Health (8 items), Social cohesion (2 items), Happiness (2 items), Feeling independent (2 items)	Internal consistency, Reliability, Content validity, Construct validity, Criterion validity, Responsiveness	Comprehensive, Profile scores, Index score, Self-administered, and Requires permission for use, Completion time (10 min)	Semantic relevance
Nottingham health profile (NHP)	Health status measure	Employment (1 item), Health (1 item), Social cohesion (6 items), Self-value (2 items), Feeling independent (2 items)	Internal consistency, Reliability, Construct validity, Responsiveness	Comprehensive, Profile scores, Index score, Self-administered, and Requires permission for use, Completion time (10 min)	Semantic relevance
Quality of life index (QLI)	Quality of life measure	Accessibility (1 item), Education (1 item), Employment (2 items), Financial stability (1 item), Health (3 items), Social cohesion (8 items), Happiness (5 items), Spirituality (1 item), Self-value (1 item), Physical appearance (2 items), Feeling independent (3 items)	Internal consistency, Reliability, Content validity, Construct validity, Criterion validity	Comprehensive, Profile scores, Index score, Self-administered, and Requires permission for use, Completion time (10 min)	Item relevance, Semantic relevance.
EuroQol-5D (EQD5)	Health status measure	Feeling independent (2 items)	Internal consistency, Reliability, Construct validity, Criterion validity, Responsiveness	Comprehensive, Index score, Self-administered, and Requires permission for use, Completion time (5 min)	Semantic relevance
Kaasa’s questionnaire (KQ)	Quality of life measure	Social cohesion (2 items), Happiness (2 items), Self-value (3 items), Feeling independent (2 items)	Internal consistency, Content validity, Construct validity	Comprehensive, Profile scores, Self-administered, and Requires permission for use, Completion time (not available)	Item relevance, Semantic relevance
Quality of life profile (QP)	Quality of life measure	Accessibility (1 item), Education (1 item), Employment (2 items), Financial stability (1 item), Social cohesion (8 items), Happiness (1 item), Self-value (1 item), Feeling independent (1 item)	Content validity	Comprehensive, Profile scores, Self/User administered, and Requires permission for use, Completion time (not available)	Item relevance, Semantic relevance
World Health Organization quality of life questionnaire- Brief (WHOQOLBREF)	Quality of life measure	Accessibility (2 items), Employment (1 item), Financial stability (1 item), Health (3 items), Social cohesion (2 items), Happiness (item), Self-value (1 item), Physical appearance (1 tem), Feeling independent (2 items)	Internal consistency, Reliability, Content validity, Construct validity, Responsiveness	Comprehensive, Profile scores, Self-administered, and Requires permission for use, Completion time (15 min)	Item relevance, Semantic relevance
Comprehensive quality of life scale (CQS)	Quality of life measure	Accessibility (2 items), Education (1 item), Employment (1 item), Financial stability (1 item), Health (2 items), Social cohesion (9 items), Happiness (2 items)	Internal consistency, Reliability, Construct validity	Comprehensive, Profile scores, User-administered, and Requires permission for use, Completion time (45 min)	Item relevance, Semantic relevance
Satisfaction with life scale (SWLS)	Life satisfaction measure	Satisfaction of needs (1 item)	Internal consistency, Reliability, Construct validity	Comprehensive, Index score, Self-administered, and Requires permission for use, Completion time (5 min)	Semantic relevance
Swedish health-related quality of life questionnaire (SWED-QUAL)	Health status measure	Health (8 items), Social cohesion (9 items), Feeling independent (8 items)	Internal consistency, Content validity, Construct validity	Comprehensive, Profile scores, Inexpensive, Self-administered, and Requires permission for use, Completion time (15 min)	Semantic relevance
Short-form 12 (SF-12)	Health status measure	Health (5 items), Social cohesion (2 items), Happiness (1 item), Feeling independent (3 items)	Internal consistency, Reliability, Construct validity, Criterion validity	Comprehensive, Profile scores, Index score, Self-administered, and Requires permission for use, Completion time (5 min)	Semantic relevance

### Intent of the instrument

Of the 11 instruments we screened, only 5 instruments were solely designed for measuring QoL. These include the Quality of Life Index (QLI), Kaasa’s Questionnaire (KQ), Quality of Life Profile (QP), World Health Organization Quality of Life Questionnaire- Brief (WHOQOLBREF), and Comprehensive Quality of Life Scale (CQS). For the remaining six instruments, five were designed to measure health status: Short-Form 36 (SF-36), Nottingham Health Profile (NHP), European Quality of Life Instrument-Five Dimensions (EQ5D), Swedish Health-related Quality of Life Questionnaire (SWED-QUAL), and the Short-Form 12 (SF-12). While the last instrument, the Satisfaction with Life Scale (SWLS), was designed to assess global satisfaction with life.

### Content suitability

In terms of content suitability, only the QLI has items that reflect all the perspectives of the polio survivors, satisfaction of needs (accessibility, education, employment and financial stability, health, and social cohesion), happiness, spirituality, and self-perception (self-value, bodily appearance, and feeling independent). This is followed by the QP, WHOQOLBREF, and CQS respectively. The QP has items reflecting most aspects of satisfaction of needs (except health) and happiness, and two aspects of self-perception, self-value and feeling independent. The items of the WHOQOLBREF reflect happiness and most aspects of satisfaction of needs and self-perception. The CQS has items reflecting the satisfaction of needs and happiness; however, the scale has no item that represents self-perception. The remaining instruments have at least one item reflecting happiness and some aspects of satisfaction of needs and self-perception. Surprisingly, none of the 11 instruments has any item that reflects spirituality except the QLI. Thus, only the QLI satisfies all the requirements of this feature.

### Measurement properties

In terms of psychometric properties, the SF-36 has good evidence of six measurement properties (internal consistency, reliability, content validity, construct validity, criterion validity, and responsiveness), followed by the QLI (internal consistency, reliability, content validity, construct validity, and criterion validity), EQ5D (internal consistency, reliability, construct validity, criterion validity, and responsiveness), and WHOQOLBREF (internal consistency, reliability, content validity, construct validity, and responsiveness) each with five measurement properties. The NHP (internal consistency, reliability, construct validity, and responsiveness) and SF-12 (internal consistency, reliability, construct validity, and criterion validity) have four adequate psychometric properties. The remaining instruments have at least three good measurement properties, except the QP, which has only evidence of adequate content validity. None of the instruments has evidence of measurement error (the systematic and random error of an individual’s score that is not attributed to true changes in the construct to be measured) or cross-cultural validity (the degree to which the performance of the items on a translated instrument are an adequate reflection of the performance of the items of the original version of the instrument). Thus, with the exception of QP, all the instruments have satisfactory evidence of good measurement properties.

### Feasibility

All the instruments are user-comprehensive. However, in terms of interpretability, only the SF-36, NHP, QLI, and SF-12 have both meaningful profile and indices scores. While the remaining instruments have either profile score or indices score. Most of the instruments can be self-administered, except the CQS, which requires administration by an interviewer. Besides, most of the instruments are available in the public domain; however, users require written permission for non-commercial use of the instruments. Most of the instruments take approximately 10 min to complete, except CQS which takes about 45 min to complete. Thus, in terms of feasibility, all the instruments are feasible for application in northwest Nigeria.

### Considerations in adapting the instrument for cross-cultural use

The QLI, KQ, QP, WHOQOLBREF, and CQS have more items that are relevant in the Hausa language compared to the remaining instruments because they are more consistent with the perspectives of polio survivors in northwest Nigeria. All the instruments have contents with semantic relevance in the Hausa language; however, some of the items of the instruments would require adaptation. Moreover, except for the WHOQOLBREF, none of the instruments have been adapted to the Hausa culture. However, the authors that translated the WHOQOLBREF did not provide information about how equivalence was established [45]. Based on the screening criteria, the contents of the QLI, KQ, QP, WHOQOLBREF, and CQS are culturally relevant in the Hausa culture.

### Selection of the instrument

**Fig. 3:**
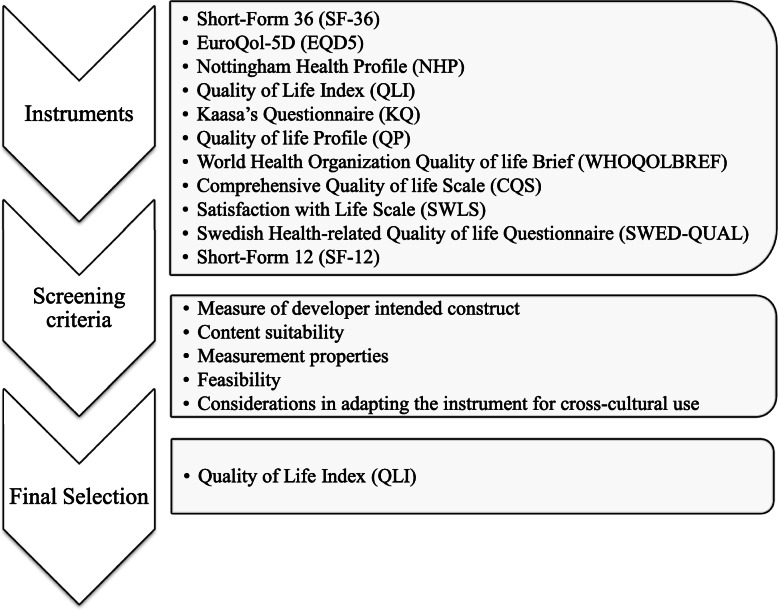
Flow diagram depicting selection of the instrument

As stated earlier, our goal was to identify and select the most appropriate QoL instrument for cross-cultural adaptation in northwest Nigeria. Specifically, we intended to select the instrument with the following features: primarily designed as a measure of QoL, consistent with the perspectives of polio survivors in northwest Nigeria, good measurement properties, feasible, and amenable to cross-cultural adaptation in northwest Nigeria. Subsequently, we identified and selected only instruments that have all or most of these characteristics. Based on the screening performance of the instruments (Table 5), the QLI has most of the required characteristics, followed by the WHOQOLBREF and CQS respectively. Hence, any of these instruments could be considered by users for cross-cultural adaption in the northwestern Nigerian context. However, the QLI appears to be more suited for the measurement of QoL of polio survivors in northwest Nigeria (Fig. 3).

The Quality of Life Index (QLI) was developed by Ferrans and Powers [19] to measure QoL in terms of life satisfaction and importance of the relevant domains of life. The instrument is made up of two parts, satisfaction and relevance, with each part containing 33 items. The items are sub-grouped into the following domains, health and functioning, social and economic, psychological and spiritual, and family. Each item is rated on a scale of one (least satisfied/important) to six (most satisfied/important). To determine the scores, each satisfaction item is weighted by a corresponding importance item. The total score of each domain ranges from 0 (less satisfied) to 30 (most satisfied) [38]. There are 14 versions of the QLI [26] and the generic and spinal cord injury versions are typically employed in polio literature [27, 54]. Although the validity and reliability of the QLI have been established, these properties are not yet reported in polio survivors [56].

Conversely, the World Health Organization Quality of Life Questionnaire-Brief (WHOQOLBREF) is an abbreviated version of the WHOQOL-100, which was developed cross-culturally by the World Health Organization Quality of life Group [59]. The WHOQOLBREF assesses QoL based on individuals’ perception of their position in life in the context of the culture and value systems in which they live and in relation to their goals, expectations, standards, and concerns [59]. Hence, the instrument provides subjective information about the individual’s life situation by taking both individual and contextual differences into account. The instrument consists of 26 items that are grouped into four domains: physical health, psychological health, social relationship, and environment [62]. To determine raw item scores, items are rated on a five-point Likert scale. Domain scores are obtained by calculating the mean score for each domain, with high scores indicating a better QoL [61]. Although WHOQOLBREF has good psychometric properties, these measurement properties were mostly established in non-polio populations such as brain injury, stroke, spinal cord injury, and Parkinson’s disease [11, 31, 33, 65]. The adapted version of the WHOQOLBREF is available in Hausa language and was tested for reliability among individuals with spinal cord injury in northern Nigeria [45]. However, the cross-cultural validity of the WHOQOLBREF was not reported and there was no clear evidence of how equivalence was attained between the original and the translated version. Hence there is a need to re-adapt the instrument using rigorous and transparent cross-cultural adaptation guidelines and replicate the already established psychometric properties of the instrument among northwest Nigerian polio survivors.

On the other hand, the Comprehensive Quality of life Scale (CQS) assesses QoL on both subjective and objective axes across seven domains, which include material well-being, health, productivity, intimacy, safety, place in the community, and emotional well-being [16]. The CQS comprises 35 items that are divided into 3 sections, objective scale, importance scale, and satisfaction scale. The objective scale has three items for each domain, while the satisfaction and importance scales have seven items each, representing the seven domains [14]. Measurement of objective QoL is achieved by obtaining an aggregate score based on the measurement of the three objective indices of each domain. While the measurement of subjective quality of life is done by obtaining a satisfaction score of each domain, which is weighted by the perceived importance of the domain to the individual [14, 16]. Domain scores are compared with normative data, with higher scores indicating a better QoL and vice versa [14, 15]. Like the QLI and WHOQOLBREF, the CQS was not evaluated for psychometric properties among polio survivors [56].

## Discussion

This review aims to identify and select a pre-existing QoL instrument that is consistent with the perspectives of polio survivors in northwest Nigeria and at the same time exhibits good psychometric properties. The findings of this review show that the Quality of Life Index (QLI), World Health Organization Quality of Life Questionnaire- Brief (WHOQOLBREF), and Comprehensive Quality of Life Scale (CQS) are consistent with the perspectives of polio survivors in northwest Nigeria and have satisfactory measurement properties [55, 56]. The QLI has most of the required characteristics, followed by the WHOQOLBREF and CQS respectively. Thus, any of these instruments could be considered by users for cross-cultural adaption in the northwestern Nigerian context. Our findings further support the application of these instruments to evaluate the QoL of polio survivors, especially in low and middle-income settings like northwest Nigeria and similar contexts.

The QLI, WHOQOLBREF, and CQS were primarily designed for the measurement of QoL and have well-established evidence of good psychometric properties [56]. In contrast with the WHOQOLBREF and CQS, the QLI has at least one item representing all the perspectives of polio survivors in northwest Nigeria. Thus, the QLI appears to be more reflective of the perspectives of the polio survivors. Other instruments screened in this review did not match the screening criteria in some critical aspects of the screening, specifically, intent of the instrument, content suitability, and considerations for cross-cultural adaptation. Hence, the instruments could not emerge as candidates of choice for cross-cultural adaptation in northwest Nigeria. One possible explanation could be because these instruments were not designed primarily to measure QoL, except the Quality of life profile (QP) and Kaasa’s Questionnaire (KQ). The QP has an insufficient record of psychometric validation [56], while the KQ was designed to assess QoL of individuals with lung cancer in clinical trials [35]. Thus, the KQ and QP could not meet the criteria for content suitability and adequate psychometric properties respectively. QoL is an elusive construct and lacks a definitive framework for conceptualization and measurement [6]. This explains why various instruments are typically employed in polio literature. We could not identify any study in the literature that integrates individuals’ perspectives to select a QoL instrument that is similar to our approach. However, most available studies typically employ psychometric properties to evaluate and select outcome measurement instruments [7, 24, 53].

To apply the QLI, WHOQOLBREF, and CQS in the measurement of QoL of polio survivors in northwest Nigeria, there is a need to cross-culturally adapt and validate the instruments in the northwestern Nigerian context [3]. Generally, cross-cultural adaptation involves translation of an instrument from a source language to target language while paying attention to any cultural difference in order to maintain equivalence in both cultures [18]. The primary aim of cross-cultural adaptation is to produce an equivalent instrument in the target culture. Five fundamental aspects of equivalence were reported in the literature, which include conceptual, item, semantic, operational, and measurement. Conceptual equivalence denotes when the domains of the instrument have the same importance and relevance in the target culture [30]. Based on our screening results, all the three prominent instruments could achieve conceptual equivalence especially the QLI, which has a complete representation of all the perspectives of the polio survivors. However, there may be some concern about the relevance of some of the items in the domains of CQS. For example, item 6a in the objective scale asks about the frequency of taking part in some leisure activities such as watching television, going to movies, hotel, pub or bar, which may not be relevant in the Hausa culture. Thus, modification of these items would be required when adapting the instrument.

On the other hand, the item equivalence refers to the acceptability of the items of the instruments in the target culture [30]. Here, there could be some cultural issues with some of the items of the instruments. For example, in the QLI and WHOQOLBREF, items 13 and 21 respectively asked about sex life, which is typically considered as an inappropriate subject in Hausa culture. Hence, modification of the items needs to be considered. Equally, semantic equivalence refers to when the items of the instruments have the same meaning in both cultures [30]. Based on the nature of the items of the instruments, some semantic issues need to be addressed when adapting the instruments. For example, two items in the QLI, “your personal appearance” and “yourself in general” could carry the same meaning in the Hausa language. Similarly, the operational equivalence is when the instruments could be employed the same way in both cultures. Since the construct ‘QoL or good life’ exist in Hausa culture, all the three instruments could be employed the same way as in their source cultures. Lastly, the measurement equivalence refers to when the instruments have the same psychometric properties in both cultures [30]. Based on the evidence of adequate psychometric properties of these instruments, there is high likelihood that they would replicate the same properties in the Hausa culture, thus attaining measurement equivalence may not be problematic.

Cross-cultural adaptation of outcome measurement instruments has many advantages over the development of a new tool. Cross-cultural adaptation is economically cheaper, less time consuming, and can produce equivalent instruments in various cultures for comparison [4, 9, 18]. Adapted instruments enable users to generalize findings across various populations and cultures, besides, users could also investigate any distinction within and between diverse population [9]. One major advantage of cross-cultural adaptation is that it is far less labor-intensive when compared to the development of new instruments [4, 18]. One caveat is that none of the instruments identified in this review are absolutely perfect matches for cross-cultural adaptation in northwestern Nigeria. Each of the selected instruments has some inherent drawbacks and may not be solely equivalent to the parent instrument when adapted to the Hausa culture. However, we believe that adapting the instruments is a more appropriate choice than creating a new one for the polio survivors in northwest Nigeria, based on the advantages of cross-cultural adaptation over the development of a new instrument.

Finally, when applying the QoL instrument for cross-cultural adaptation, psychometric validation, or routine QoL evaluation, users should take response shift into consideration, in order to avoid paradoxical scores of QoL measurement. As pointed out earlier in the introduction, response shift can occur as a result of redefinition of the construct, when the individual readjust their priorities as a result of a life-changing event, environmental influences, and newly acquired coping strategies. Moreover, response shift could also ensue when the individual reprioritizes their values or change their internal standards of measurement through recalibration [49]. Various protocols for addressing response shift when evaluating QoL among people with chronic conditions were proposed and applied in the literature such as the then-test and structural equation modeling [20, 22, 50]. These techniques could be employed when measuring the QoL of polio survivors in northwest Nigeria as well.

### Strengths and limitations

We could not identify any study in the literature that integrates individuals’ perspectives to select a QoL instrument that is similar to our process. Our approach to the selection of QoL instrument is based on rigorous recommendations and the perspectives of polio survivors (in northwest Nigeria), to select the most appropriate instrument available. Moreover, we considered critical aspects for cross-cultural adaptation and feasibility of the instrument in addition to psychometric properties. However, despite our effort to ensure rigor, this review has some limitations. For example, we did not assess client comprehensibility of the instruments, which could compromise possible self-administration of the instrument. Moreover, because our study was based on a previous scoping review, we may have not included other relevant QoL assessment instruments in our screening.

### Conclusion and recommendations

Most instruments that are used to assess the QoL of polio survivors were not primarily designed for the measurement of QoL. Thus, it is pertinent to employ instruments that are specifically designed for QoL evaluation. To select a contextually relevant instrument, there is a need to consider and integrate the perspectives of the individuals. Our findings show that the QLI, WHOQOLBREF, and CQS are consistent with the perspectives of polio survivors in northwest Nigeria. Hence, these instruments could be used to evaluate the QoL of polio survivors in northwestern Nigeria and similar contexts. Although the instruments have demonstrated good measurement properties, we recommend the validation of the instruments among Nigerian polio survivors. Drawing upon the screening performance of the instruments, the QLI exhibits the following characteristic features: primarily designed as a measure of QoL, more consistent with the perspectives of polio survivors in northwest Nigeria, more evidence of good measurement properties, feasible, and amenable to cross-cultural adaptation in northwest Nigeria. Hence, we recommend the translation, cross-cultural adaptation, and psychometric validation of the QLI among polio survivors in northwest Nigeria. A rigorous protocol of cross-cultural adaptation such as the International Society for Pharmacoeconomics and Outcomes Research principles of good practice can be considered. Measurement of QoL among polio survivors in northwest Nigeria could provide the impetus needed to draw the attention of stakeholders toward addressing the unmet needs of the individuals. Therefore, to generate high-quality evidence about the QoL of the polio survivors, a culturally relevant and valid instrument is necessary; which researchers can produce using cross-cultural adaptation and psychometric validation techniques.

## Data Availability

Not applicable.
